# Functional and Structural Abnormalities in Deferoxamine Retinopathy: A Review of the Literature

**DOI:** 10.1155/2015/249617

**Published:** 2015-06-08

**Authors:** Maura Di Nicola, Giulio Barteselli, Laura Dell'Arti, Roberto Ratiglia, Francesco Viola

**Affiliations:** ^1^Ophthalmological Unit, Department of Clinical Sciences and Community Health, Ca' Granda Foundation-Ospedale Maggiore Policlinico, University of Milan, Via Francesco Sforza 35, 20122 Milan, Italy; ^2^Genentech, Inc., 1 DNA Way, South San Francisco, CA 94080, USA

## Abstract

Deferoxamine mesylate (DFO) is the most commonly used iron-chelating agent to treat transfusion-related hemosiderosis. Despite the clear advantages for the use of DFO, numerous DFO-related systemic toxicities have been reported in the literature, as well as sight-threatening ocular toxicity involving the retinal pigment epithelium (RPE). The damage to the RPE can lead to visual field defects, color-vision defects, abnormal electrophysiological tests, and permanent visual deterioration. The purpose of this review is to provide an updated summary of the ocular findings, including both functional and structural abnormalities, in DFO-treated patients. In particular, we pay particular attention to analyzing results of multimodal technologies for retinal imaging, which help ophthalmologists in the early diagnosis and correct management of DFO retinopathy. Fundus autofluorescence, for example, is not only useful for screening patients at high-risk of DFO retinopathy, but is also a prerequisite for identify specific high-risk patterns of RPE changes that are relevant for the prognosis of the disease. In addition, optical coherence tomography may have a clinical usefulness in detecting extent and location of different retinal changes in DFO retinopathy. Finally, this review wants to underline the need for universally approved guidelines for screening and followup of this particular disease.

## 1. Introduction

Deferoxamine mesylate (DFO) is the most used iron-chelating drug to treat hemosiderosis secondary to transfusions. Deferoxamine mesylate is most commonly administered as a slow subcutaneous infusion but can also be given intramuscularly or, less commonly, intravenously [1]. Long-term treatment with blood transfusions effectively prevents various complications of sickle cell anemia and can sustain patients with chronic congenital and acquired refractory anemia, including beta-thalassemia syndromes, myelodysplastic syndromes, myelofibrosis, aplastic anemia, and other disorders [1]. The use of iron-chelating agents is crucial for the management of such diseases. Since the human body has no physiologic mechanisms to discard excess iron [2], the frequent blood transfusions required in these conditions inevitably produce iron overload. If not treated, chronic iron overload can result in multiple organ toxicities including potentially fatal cardiac toxicity, hepatic fibrosis or cirrhosis, impaired growth, failure of sexual maturation, and diabetes [3]. In patients with thalassemia who undergo transfusion from infancy, iron-induced liver disease and endocrine disorders develop during childhood and are almost inexorably followed by death from iron-induced cardiomyopathy in adolescence [1]. Deferoxamine mesylate has also been used for the treatment of acute iron intoxication and as a screening test for increased aluminum body stores in chronic renal failure [4, 5]. Deferoxamine mesylate has high affinity for ferric iron, thus removing iron from hemosiderin, ferritin, and transferrin [6].

## 2. Complications of Deferoxamine Mesylate Therapy

### 2.1. Side Effects

Despite the clear advantages for the use of DFO, numerous significant drug-related toxicities have been reported in the literature. Systemic toxicities included cardiovascular, respiratory, gastrointestinal, cutaneous, and nervous systems [1], in addition to the propensity for bone dysplasia [7] and high-frequency sensorineuronal hearing loss [8–10]. Furthermore, DFO therapy may induce ocular toxicity consisting of retinal pigment epithelium (RPE) changes, visual loss, and impaired night vision [6, 11]. As a result, the damage to the RPE can lead to visual field defects, decreased visual acuity, color-vision defects, and decreased responses during electroretinogram (ERG) and electrooculogram (EOG) [11–13]. Since the 1980s, several case reports and small case series have been reported confirming these findings, which may develop not only after high-dose intravenous but also after subcutaneous administration (Table 1) [6, 9–30]. Varying conclusions have been reported regarding reversibility of DFO toxicity [26]; while usually visual deficits recover after cessation of the medication [15, 31], some authors reported permanent visual deterioration [13] or even progression of the retinopathy even after DFO discontinuation [29, 32].

### 2.2. Mechanism of Toxicity

The mechanism of DFO toxicity has been extensively studied; however, it is still not well understood. Rahi et al. were the first to perform a histologic and ultrastructure examination of an eye diagnosed with DFO retinopathy [16]. They reported abnormalities that resembled apoptotic changes of the RPE. These changes included patchy depigmentation in the equatorial as well as the posterior fundus, swelling and calcification of mitochondria, disorganization of the plasma membrane, loss of microvilli from the apical surface, and vacuolation of the cytoplasm. RPE cells appeared enlarged and projected into the subretinal space, which sometimes showed detached and rounded RPE cells containing typical melanin accumulation. Thickening of Bruch membrane overlying the RPE was noted as well.

It is also known that administration of DFO results in high fecal iron, copper, and zinc excretion due to the drug's chelation properties [33]. De Virgiliis et al. hypothesized that DFO retinopathy may be related to either serum or intracellular zinc and copper deficiencies [33]. Indeed, zinc or zinc compounds are known to enhance the antioxidative capability of RPE cells [34, 35]. Pall et al. hypothesized that DFO retinopathy may occur in situations where the dose of DFO is too high compared to the stores of iron present [36]. In these situations, the excess DFO could bind copper and result in copper-induced autooxidative damage.

More recently, Klettner et al. examined the direct toxic effect of DFO on cultured primary RPE cells [37]. These investigators were the first to clarify in vitro that the toxic effect of DFO to the RPE is direct and not secondary to trace element depletion. In addition, they showed that the cell death was mediated by the activation of p38 mitogen-activated protein kinases. These protein kinases were previously shown to be important for the execution of programmed cell death after toxic stimuli [26]. Finally, the investigators indicated a general involvement of p38 in stress-induced cell death in RPE cells, since p38 is also involved in oxidative stress.

### 2.3. Incidence

There are different estimates of the incidence of retinal toxicity due to DFO. Olivieri et al. reported that 5.6% of patients receiving DFO therapy had RPE changes [9]. In a series of 52 regularly transfused patients who received DFO by subcutaneous or intravenous infusion, Cohen et al. found that only two patients had abnormal visual screening tests; one of them was symptomatic and one was not [10]. Chen et al. reported on a series of 30 transfusion-dependent patients receiving DFO in a dose of 40 to 50 mg/kg subcutaneously overnight for 8 to 10 hours by pump, 4 to 7 days per week, and detected no visual abnormality [8]. In another series of 84 children with transfusional hemochromatosis, drug-related ocular toxicity was found only in one patient (1.2%) [26]. Finally, our group has recently reported on a large series of 197 consecutive adult patients with beta-thalassemia syndromes receiving chronic treatment with DFO and found abnormal fundus autofluorescence (FAF) which is not related to other diseases in 9% of the patients [29].

### 2.4. Risk Factors

A clear relationship between drug dosage and development of DFO retinopathy could not be identified in most case series [6, 14]. Previously cited risk factors for visual loss in DFO retinopathy included blood-retinal barrier breakdown associated with diabetes [14] and rheumatoid arthritis [15], renal failure [13], and metabolic encephalopathy [15]. It is believed that blood-retinal breakdown, which can be due to iron overload-induced diabetes or to the drug itself, may increase DFO levels in the RPE and retina, thus intensifying the local toxic effects of the drug [6]. It has also been suggested that older age and longer duration of DFO treatment may be associated with more advanced forms of retinopathy in patients with beta-thalassemia syndromes [32, 38].

## 3. Clinical Presentation

Previously reported ocular findings of DFO toxicity include cataract, optic neuropathy, optic atrophy, and macular or equatorial pigmentary degeneration [6, 9, 10, 12, 15, 33]. On fundus examination, the acute stage of DFO retinopathy is characterized by retinal opacification or loss of transparency, as well as EOG and ERG attenuation [6]. This stage is followed by macular and/or equatorial RPE pigmentary mottling, which persists even after functional recovery. Multiple case reports have described characteristic fundus lesions of DFO retinopathy seen by ophthalmoscopy and fundus photography; these lesions included pigmentary retinopathy, bull's eye maculopathy, and vitelliform maculopathy [15, 19, 21, 25, 27]. However, the use of high-resolution imaging technologies has demonstrated that DFO retinopathy may present ophthalmoscopically with a variety of RPE degenerative patterns resembling pattern dystrophies or may present with only minimal changes in the macula that can be easily missed by indirect ophthalmoscopy [29]. Multimodal imaging using confocal laser scanning ophthalmoscopy (cSLO) was shown to be extremely useful in detecting early RPE changes related to DFO retinopathy, as well as in analyzing longitudinal modifications of the disease.

## 4. Tests for Functional Abnormalities

### 4.1. Electrophysiology

Electrophysiological tests have been widely used to establish the diagnosis of DFO retinopathy. They are essential for gathering information on the location and extent of retinal dysfunction in patients with this pathology. The degree of functional loss in DFO retinopathy can be assessed by using ERG or also EOG. Electrophysiology is a valuable tool that is usually confirmatory for the diagnosis, and sometimes may also indicate more widespread dysfunction than may be implied by funduscopy alone. Electrophysiology performed in rats given intravenous DFO showed early dose-related suppression of b-wave amplitude [39, 40] that in some cases were reversible [41]. Arden et al. studied 43 patients with thalassemia major and intermedia requiring regular blood transfusions without any ocular symptoms. They found that while EOG and ERG results where only slightly and insignificantly lower than average, pattern ERG abnormalities were much more pronounced [14]. Multifocal ERG was found to be helpful as well in demonstrating areas of decreasing function over time in DFO maculopathy [23]. A longitudinal ERG study in 11 beta-thalassemia major patients receiving DFO suggested scotopic dysfunction, most likely related to iron toxicity [42]. Lakhanpal et al. used electrophysiological tests to study eight patients who developed ocular toxicity while undergoing DFO therapy for transfusional haemosiderosis and suggested toxicity at the level of the RPE and photoreceptors [12]. Haimovici et al. performed electrophysiological tests on 16 patients with visual loss and macular pigmentary changes related to DFO retinopathy. They found reduced ERG amplitudes and reduced EOG light-peak to dark-trough ratios indicating retinal and RPE injury [6]. Electrophysiological results may also normalize after splenectomy and cessation of DFO therapy [22].

### 4.2. Visual Field

Several case reports and case series reported on visual field alterations in patients undergoing long-term DFO treatment. The most common alterations were a generalized constriction of the visual field [14, 28] or central-paracentral scotomata [17, 25, 27]. Rahi et al. reported a case that presented with both central scotoma and constriction of the peripheral field in each eye [16]. These abnormalities resolved after withdrawal of high-dose therapy.

### 4.3. Microperimetry

**Figure 1:**
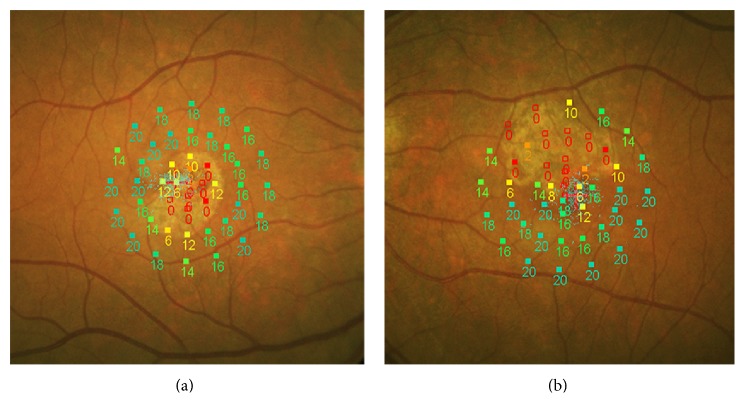
Microperimetric results in two eyes with DFO retinopathy. Absolute scotomata are present in macular areas of RPE atrophy as seen on fundus photography. Relative scotomata or reduced retinal sensitivity are present in the adjacent areas where RPE changes may or may not be visible.

To date, microperimetric results in DFO retinopathy have not been reported in the literature. However, microperimetry can be a useful tool to study the impact of macular RPE changes on visual function in this disease. The latest models of microperimeters incorporate a color fundus camera for image registration and an autotracking system to facilitate the accurate measurement of retinal sensitivity within the central visual field, even in patients with unstable or extrafoveal fixation. This allows detection of absolute scotomata, relative scotomata, or abnormally reduced retinal sensitivity in patients with macular pathologies. Examples of microperimetric results in eyes with DFO retinopathy are shown in Figure 1.

## 5. Tests for Structural Abnormalities

### 5.1. Fluorescein Angiography

**Figure 2:**
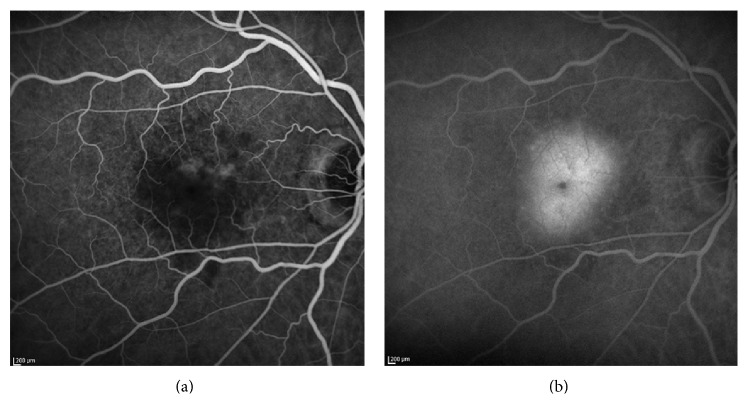
Early (a) and late phase (b) fluorescein angiography in an eye with DFO retinopathy. The angiogram showed patchy blocked fundus fluorescence in early phase in the macula, followed by late staining.

In the past, fluorescein angiography (FA) has been widely used to diagnose DFO retinopathy [6, 17, 20, 21, 23, 27, 28]. In the earliest stages, when ophthalmoscopy shows loss of retinal transparency only, FA shows patchy blocked fundus fluorescence followed by late staining (Figure 2). When pigment mottling develops, FA shows mottled fluorescence in the early-phase angiogram with late hyperfluorescence [6]. However, these FA findings are not pathognomonic of DFO retinopathy. With the advent of noninvasive high-resolution imaging technologies such as the cSLO, FA is now only rarely required for the diagnosis of patients with DFO retinopathy.

### 5.2. Fundus Autofluorescence on Confocal Scanning Laser Ophthalmoscopy

To date, FAF imaging using cSLO seems to be the most effective clinical adjunct for the diagnosis and evaluation of patients with DFO retinopathy [29]. The FAF signal generally provides indirect information on the level of metabolic activity of the RPE, since it represents an index of lipofuscin accumulation [43]. Fundus autofluorescence appearance of a normal fundus is characterized by a homogeneous background autofluorescence arising from the RPE, with a gradual decrease in macular FAF intensity towards the foveola that results from the masking effect of yellow macular pigment. This suggests that, in vivo, FAF imaging may represent a suitable noninvasive diagnostic tool to detect early RPE abnormalities in various retinal disorders, including drug-related retinal toxicity [44]. Indeed, it has been shown that FAF imaging is superior to ophthalmoscopy in detection of early characteristic RPE abnormalities in patients at risk of DFO retinopathy, as well as in monitoring the disease progression over time [29].

In 2012, our group described a variety of phenotypic patterns of abnormal FAF in thalassemic patients who needed long-term DFO treatment with the use of a cSLO device [29]. The topographic FAF alterations were classified into four different patterns using a slightly modified classification for age-related macular degeneration published by the FAM Study group [45]. The characteristics of each pattern are described below.

**Figure 3:**
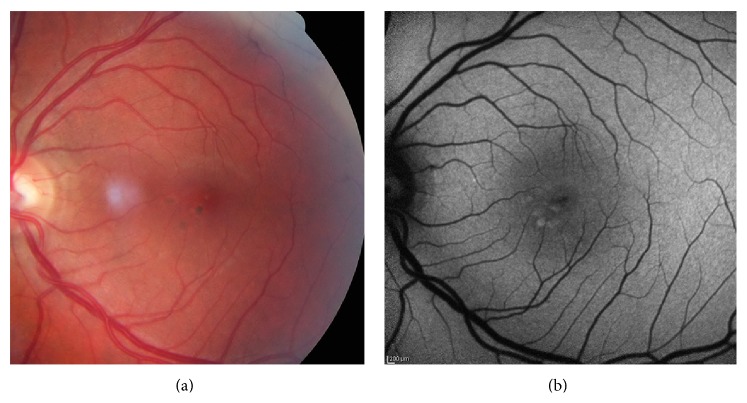
Color photo (a) and FAF image (b) of a minimal change pattern of DFO retinopathy.

*(1) Minimal Change Pattern (Figure 3).* Eyes with only minimal variations from the normal FAF appearance, with irregularly increased or decreased background FAF, are included in this group. Increased FAF signal, which may result from mottling of the RPE, is characterized by relatively small spots of less than 100 microns in diameter within the macula. The spots have well-defined borders and in some cases correspond to visible alterations on color fundus photographs, such as focal hyperpigmentation.

**Figure 4:**
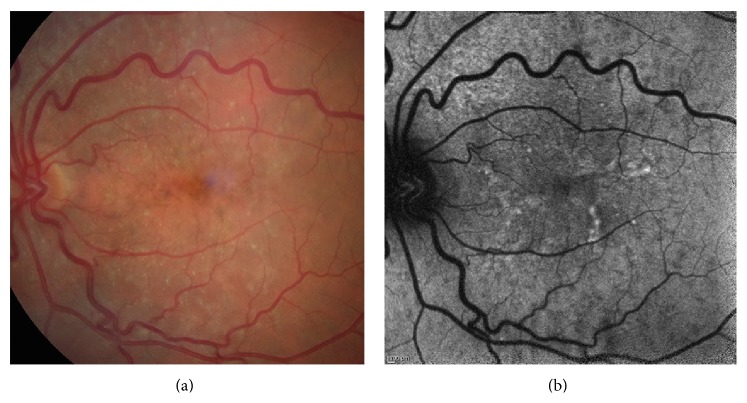
Color photo (a) and FAF image (b) of a focal pattern of DFO retinopathy.

*(2) Focal Pattern (Figure 4).* This pattern is defined by the presence of at least one medium-sized spot, more than 100 microns but less than 200 microns in diameter, of markedly increased FAF that is much brighter than the surrounding background FAF. The borders appear well-defined, with no gradual decrease of FAF observed between the background and the area with focally increased FAF. On color fundus photographs these spots may correspond to visible alterations, such as focal hyperpigmented areas.

**Figure 5:**
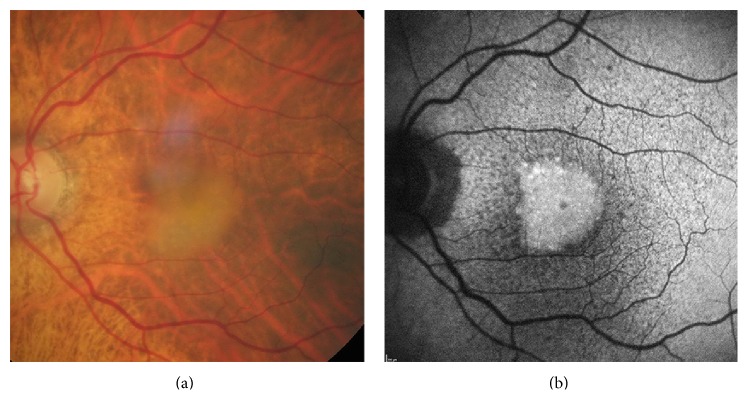
Color photo (a) and FAF image (b) of a patchy pattern of DFO retinopathy.

*(3) Patchy Pattern (Figure 5).* This pattern is characterized by the presence of at least one large area, more than 200 microns in diameter, of markedly increased FAF. These areas are brighter than the surrounding background FAF, usually with well-defined borders. Nevertheless, coalescence of these areas usually occurs, resembling a pattern dystrophy. The corresponding abnormalities are visible on color fundus photographs and include both hyperpigmentation and hypopigmentation. To note, the affected area can often appear larger in FAF imaging than that expected from the color fundus photographs and sometimes it includes different intensities of hyperautofluorescence.

**Figure 6:**
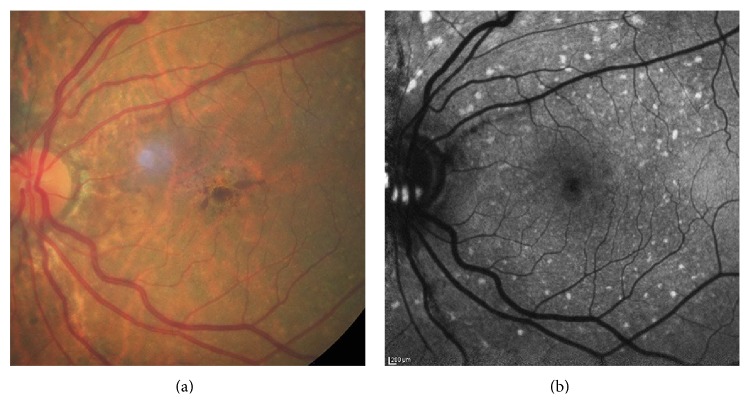
Color photo (a) and FAF image (b) of a speckled pattern of DFO retinopathy.

*(4) Speckled Pattern (Figure 6).* The speckled pattern is defined by the simultaneous presence of a variety of FAF changes that extend beyond the macula. Typically, these abnormalities include multiple small areas of irregularly increased and decreased FAF. On color fundus photographs, these abnormalities sometimes correspond to visible alterations such as focal hyperpigmentation and hypopigmentation. The pathologic areas seem to be fewer and smaller than the corresponding color fundus photographs.

As reported by our group, the detected FAF alterations in DFO retinopathy were always bilateral but asymmetrical [29]. The most frequent pattern was the minimal change pattern (56%), followed by the focal pattern (17%), the patchy pattern (16%), and the speckled pattern (11%). No association was found between pattern type and duration of DFO treatment. Areas of increased FAF signal indicated diffuse accumulation of autofluorescent fluorophores within a thickened RPE-Bruch membrane complex or also focal accumulation of autofluorescent outer segment-derived retinoid products in the subretinal space. The different intensities of hyperautofluorescence could be related to the presence of various materials within different retinal locations.

**Figure 7:**
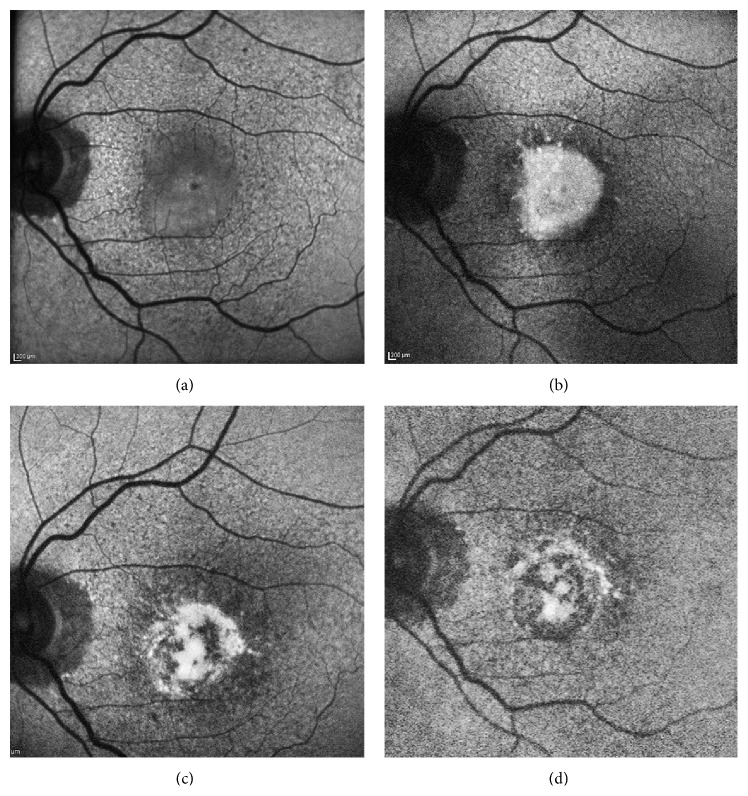
Serial fundus autofluorescence (FAF) images of a patient with patchy pattern during a 5-year follow-up. (a) Presence of a patchy area with mildly increased FAF in the inferior macula at baseline examination, involving the fovea. (b) At year 2, the patchy area showed a much greater and uniform increased FAF signal compared to the previous visit. (c) At year 4, part of the patchy area of increased FAF signal started disappearing, and areas of retinal pigment epithelium atrophy started developing. (d) At year 5, most parts of the patchy area of increased FAF signal shrunk and disappeared, leading to frank retinal pigment epithelium atrophy in the macula.

**Figure 8:**
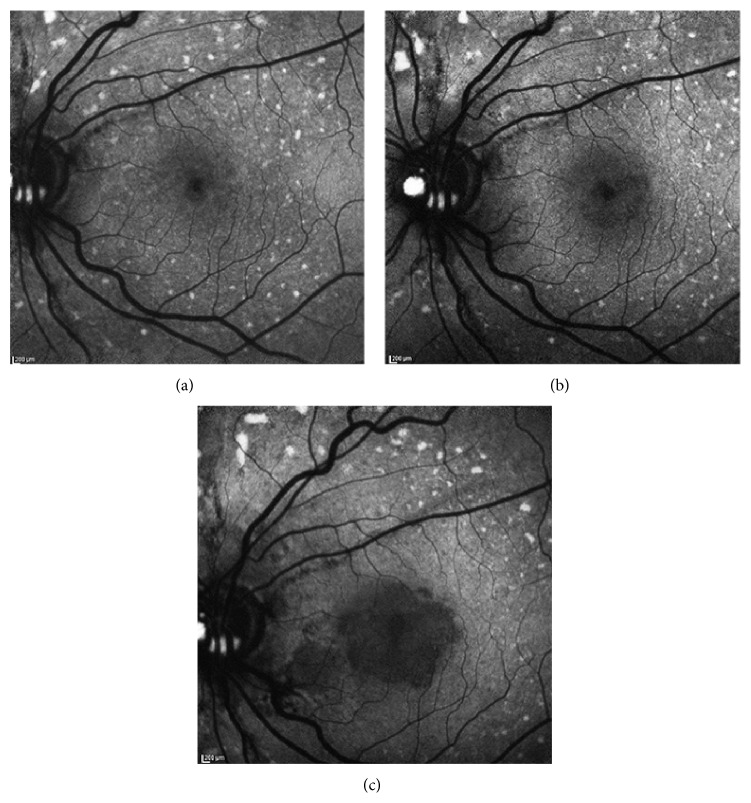
Serial fundus autofluorescence (FAF) images of a patient with speckled pattern during a 3-year follow-up. (a) At baseline examination, multiple granular spots of increased FAF were clearly detected in the macula and also beyond the vascular arcades. In the perifoveal area the spots were partially confluent. (b) After 1 year, the perifoveal spots began to progressively disappear and initial RPE atrophy occurred. (c) At year 3, further enlargement of the RPE atrophy in the macula as well as further reduction of the perimacular spots of increased FAF signal were clearly detected.

Besides being extremely helpful for detecting early RPE changes, FAF has been shown to be very useful in evaluating the clinical course of DFO retinopathy as well. To date, the longest average follow-up of cases of DFO retinopathy using multimodal imaging including FAF is 20 months (range: 10 to 45 months) [32]. In cases of minimal changes in the macula, a slight enlargement of the affected areas developed over the course of the years if DFO was not discontinued. Limited FAF changes were detected in eyes with focal pattern, independently from the ongoing or discontinued DFO treatment. In cases of patchy pattern (Figure 7) or speckled pattern (Figure 8), follow-up examinations revealed progressive development of RPE atrophy in the previously affected hyperautofluorescent areas. In addition, RPE atrophy progressively enlarged during the ensuing visits, leading to irreversible vision loss. Notably, none of the patients with patchy pattern could discontinue DFO treatment due to their precarious systemic conditions [29, 32].

Patients with minimal changes in the macula were found to be younger than patients with the other patterns [32]. It was therefore hypothesized that minimal changes related to DFO retinopathy may progress into other patterns with increasing age of the patients. However, a longitudinal study with longer follow-up duration is necessary to detect a significant disease progression from one pattern to another.

### 5.3. Spectral Domain Optical Coherence Tomography

**Figure 9:**
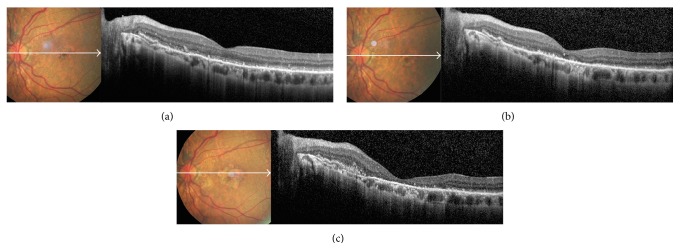
Serial color photos and OCT scans of a patient with speckled pattern during a 3-year follow-up. (a) At baseline examination, pigmented material was visible in the fovea, with small yellow flecks extending beyond the vascular arcades. On OCT scan, granular hyperreflective deposits were detected in the subretinal space, extending into the outer plexiform layer and interrupting the overlying external limiting membrane. (b) After 1 year, initial RPE atrophy was visible around the fovea, with disruption of the outer retinal layers on OCT scan. (c) At year 3, frank RPE atrophy developed in the macula; OCT scan showed atrophy of the outer retinal layers and RPE, as well as thinning of the inner retina.

**Figure 10:**
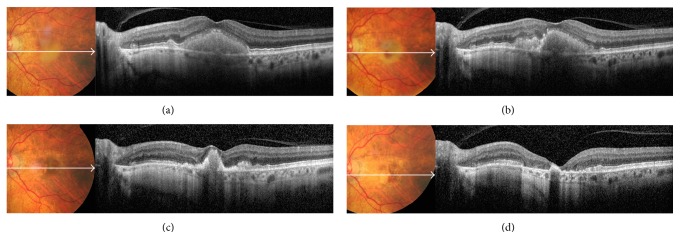
Serial color photos and OCT scans of a patient with patchy pattern during a 5-year follow-up. (a) At baseline examination, yellowish vitelliform-like material was visible on fundus photo; OCT scan showed that the material was homogeneous, mildly hyperreflective, and localized in the subretinal space above the RPE. It was associated with a diffusely thickened inner segment/outer segment junction and intact external limiting membrane. (b) After 1 year, the vitelliform material started resorbing inferonasally to the fovea; on OCT scan, the subretinal material was no more homogeneous. (c) At year 3, the vitelliform material shrunk, and initial RPE and photoreceptors atrophy were appreciated perifoveally. (d) At year 5, the vitelliform material was completely resorbed; OCT scan showed absence of external limiting membrane, marked thinning of the outer nuclear layer, and frank RPE and photoreceptors atrophy in the macula.

Spectral domain optical coherence tomography (SD-OCT) may have a clinical usefulness in detecting extent and location of the different retinal degenerations in DFO retinopathy [30, 32]. Some SD-OCT devices can also couple OCT scan and bidimensional cSLO image to simultaneously colocalize posterior structures with high accuracy [46]. In early stage of the disease, SD-OCT usually shows only focal thickenings or bumps of the RPE, resembling basal laminar drusen. As the disease progresses, the coalescence of these bumps of pigmented material appears on SD-OCT as thick and hyperreflective dome-shaped lesions that disrupts the architecture of the overlying outer retinal layers. As the pigmented material reabsorbs, RPE becomes progressively thinner (Figure 9). Sometimes vitelliform-like material could develop in the subretinal space above the RPE (Figure 10), associated with a diffusely thickened inner segment/outer segment junction. In advanced stages of DFO retinopathy, frank RPE and photoreceptors atrophy may develop in the macula, as well as migration of hyperreflective subretinal deposits towards the outer plexiform layer interrupting the overlying external limiting membrane [32].

## 6. Management

Currently, there are no approved guidelines for the screening and follow-up of DFO retinopathy in patients requiring regular blood transfusions. Also, there is no treatment available for patients with DFO retinopathy other than drug discontinuation or dose reduction. To minimize risks of DFO retinopathy, it has been suggested to not exceed doses of 50 mg/kg of body weight in patients with iron overload and to decrease the dose as the hepatic iron concentration approaches normal levels [1]. Although treatment with DFO may reduce endocrine complications of iron overload, such as a delay of puberty, the chelator itself can interfere with growth [47], apparently as a result of skeletal dysplasia [48]. To minimize this effect, the dose of DFO in children should not exceed 25 to 30 mg/kg [49]. Since ocular changes related to DFO toxicity are potentially sight-threatening, we believe that regular ocular checkups are essential for patients undergoing treatment. The presence of minimal changes in the macula can be followed every 6 months, but patients with certain toxicity such as bilateral pattern dystrophy-like changes should stop the drug immediately unless the risks of their underlying disease outweigh the risks of permanent and possibly progressive visual loss. Even after cessation of the drug, we recommend patients to return for reexamination every 3 months.

New iron-chelating therapies (deferasirox and deferiprone) are now available in the market, but their long-term ocular safety has not been comprehensively investigated. Moreover, the data considering side effects of deferiprone and deferasirox are controversial [50, 51], yet there are studies in which deferiprone seems to display fewer side effects than DFO [52]. In particular, deferasirox could cause potentially fatal renal and hepatic impairment or failure as well as gastrointestinal hemorrhage [1]. These adverse effects were reported to occur more frequently in older patients and in patients with high-risk myelodysplastic syndromes, thrombocytopenia, or underlying renal or hepatic impairment. Deferiprone could cause diarrhea and gastrointestinal effects, arthropathy, increased levels of serum liver enzymes, and progression of hepatic fibrosis associated with an increase in iron overload or hepatitis C. The most serious adverse effects are agranulocytosis and neutropenia; weekly monitoring of the neutrophil count is recommended [1].

## 7. Conclusion

Deferoxamine mesylate is the most important drug for the treatment of hemosiderosis secondary to long-term treatment with blood transfusions. Many different ocular toxicities have been reported in the literature, with the most serious being sight-threatening retinopathy. Currently, no “gold standard” exists for identification of the ocular toxicity prior to its development. This has led to the importance of repeated ophthalmologic examinations for screening patients. With the development of high-resolution noninvasive imaging technologies, we believe that FAF can be used as a rapid and reliable way to evaluate DFO retinopathy. Fundus autofluorescence imaging on a cSLO device not only is useful for screening patients at high-risk of the disease, but it also allows longitudinal evaluation of eyes with DFO retinopathy. These changes are more widespread on FAF imaging than expected from fundoscopy, and DFO retinopathy may also present with different FAF patterns that are relevant for the prognosis of the disease. Fundus autofluorescence imaging is a prerequisite for identifying specific high-risk characteristics (such as patchy or speckled patterns) that may be helpful in the decision to discontinue or switch iron-chelating therapy to prevent disease progression and irreversible visual loss due to RPE atrophy. In addition to FAF imaging, SD-OCT may have a clinical usefulness in detecting extent and location of different retinal changes in DFO retinopathy. Further, longitudinal, multicenter studies with longer follow-up and larger population may clarify any relationship with the onset of a particular FAF pattern, any progression from one pattern to another, and whether or not retinal alterations have functional (e.g., localized scotomas) and/or prognostic (e.g., on the development of choroidal neovascularization) consequences.

## Figures and Tables

**Table 1:** Summary of cases with DFO retinopathy in the literature.

Author, year	Patients with DFO retinopathy	DFO administration	DFO duration	Retinal findings (patients)	Elettrophysiology findings (patients)	Visual field findings (patients)
Davies et al., 1983 [11]	2	IV	—	—	ERG: abnormal	—
Arden et al., 1984 [14]	18	SC	4 to 5 years	RPE hyperpigmentation	EOG: subnormal (12); ERG: reduced cone function (7); PERG: reduced responses (18)	Visual field constriction
Lakhanpal et al., 1984 [12]	7	IV	—	Macular and peripheral RPE mottling	—	—
Blake et al., 1985 [15]	3	IV	5 to 12 days	Pigmentary retinopathy and macular RPE mottling	EOG: abnormal; ERG: subnormal	—
Rahi et al., 1986 [16]	1	IM and then SC and then IV	24 years	—	ERG: subnormal; EOG: abnormal	Visual field constriction and central scotoma
Olivieri et al., 1986 [9]	5	SC	—	Macular RPE mottling	—	—
Bene et al., 1989 [13]	1	—	—	—	—	—
Ravelli et al., 1990 [17]	5	IV	5 months	Macular RPE mottling (4)	EOG: abnormal	Central scotoma (1)
Cohen et al., 1990 [10]	2	—	—	Macular RPE mottling (1)	—	—
Mehta et al., 1994 [18]	1	SC	—	—	—	—
Haimovici et al., 2002 [6]	16	SC (5) and IV then SC (2), IV (1), IP (1), and NK (7)	1 month to 10 years	RPE opacification (5) and RPE mottling (14)	ERG: reduced cone and rod function; EOG: abnormal	—
Bansal et al., 2003 [19]	1	—	26 years	Bull's eye maculopathy	—	—
Kertes et al., 2004 [23]	1	SC	18 months	Macular RPE mottling	Multifocal ERG: reduced responses	—
Arora et al., 2004 [20]	1	IV	3 years	Macular RPE mottling	ERG: normal rods responses, reduced cone responses	—
Hidajat et al., 2004 [22]	1	SC	2 years	Macular RPE mottling	EOG: flat or subnormal	—
Gonzales et al., 2004 [21]	2	SC (1) and IV (1)	11 months (SC); 3 years (IV)	RPE mottling (1) and vitelliform lesion (1)	ERG: reduced cone responses; EOG normal (1)	—
Lai et al., 2006 [24]	1	SC and then IV	14 years (SC); 4 months (IV)	Macular and peripheral RPE mottling	ERG: reduced cone and rod responses; EOG: abnormal	—
Lu et al., 2007 [25]	1	SC	36 years	Pigmentary retinopathy	ERG: reduced rod responses	Central, paracentral scotoma
Baath et al., 2008 [26]	1	SC	1 to 17 years	Macular RPE mottling	ERG: reduced rod responses	—
Genead et al., 2010 [27]	1	IM and then SC	20 years	Vitelliform lesion and RPE mottling	ERG: normal cone and rode function	Central scotoma
Simon et al., 2012 [28]	1	SC and then IV	23 years (SC); 10 weeks (IV)	Macular RPE mottling	ERG: reduced cone and rod responses; EOG: abnormal	Visual field constriction
Viola et al., 2012 [29]	18	SC	10 to 55 years	Pattern dystrophy-like changes (8) and minimal RPE mottling (10)	—	—
Wu et al., 2014 [30]	1	SC and then IV	20 years (SC); 42 days (IV)	Macular and peripheral RPE mottling	—	—

DFO: deferoxamine; RPE: retinal pigment epithelium; IV: intravenous; SC: subcutaneous; IP: intraperitoneal; IM: intramuscular; NK: unknown; ERG: electroretinogram; EOG: electrooculogram.
